# Healing of a large skin defect in a dog with concurrent ozonated olive oil application

**DOI:** 10.1111/jsap.13477

**Published:** 2022-01-18

**Authors:** P. Prządka, M. Kuberka, P. Skrzypczak, Z. Kiełbowicz

**Affiliations:** ^1^ Department and Clinic of Surgery, Faculty of Veterinary Medicine Wroclaw University of Environmental and Life Sciences 50‐366 Wroclaw Poland; ^2^ Veterinary Clinic Kuberwet 63‐300 Pleszew Poland

**FIG 1:**
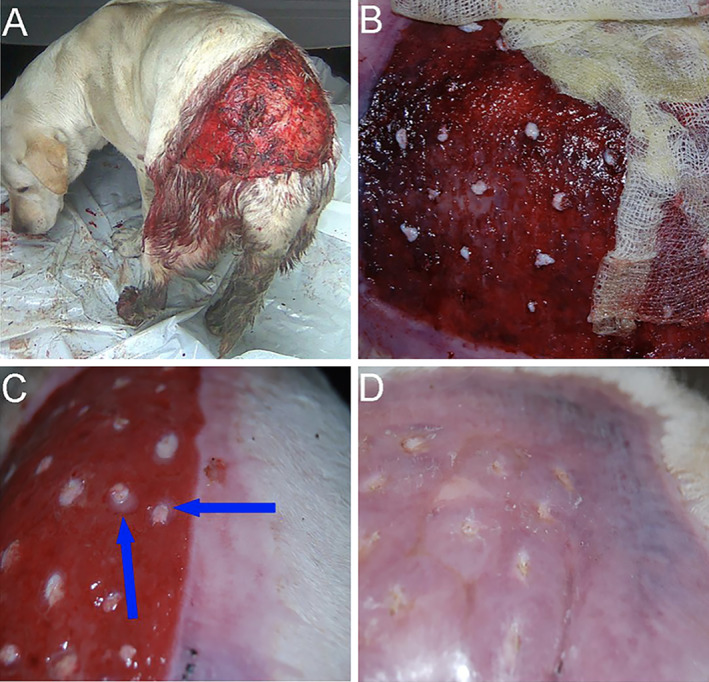
Stages of treatment of a large skin wound with a skin graft using the pinch grafts technique, protected with a dressing soaked in ozonated oil. (A) The appearance of the wound before treatment. (B) The appearance of skin grafts on the third day after surgery including gauze soaked in ozonated oil – old dressing during removal. (C) The appearance of skin grafts on day 12 after transplantation. At the periphery of the skin grafts, there is beginning epidermal growth covering the wound (blue arrows). (D) Appearance of the wound at day 35, completely covered with epithelium

A 3‐year‐old, entire female Labrador retriever, was admitted with an extensive full thickness skin laceration in the lumbar region (Fig 1A). The injury was surgically debrided. The wound was covered with a non‐adherent moist dressing. The layer in contact with the wound was made of surgical swabs soaked in 20 mL ozonated olive oil (Ozonella, Onkomed, Poland). The dressing was replaced every 48 hours. On day 12 after the injury, a skin transplant was performed. Pinch grafts obtained from the medial side of the thighs were transplanted onto the granulating tissue of the healing wound (Fig 1B). The wound was covered again with a non‐adherent moist dressing composed of surgical swabs soaked in ozonated olive oil. The swabs were kept in place by absorbent cotton roll and then self‐adherent elastic wrap. The dressing was replaced every 24 to 72 hours. No antibiotics were used during whole treatment of the described case. Ninety‐two percent of the transplanted fragments healed into the recipient site (Fig 1C). Throughout the post‐transplant period, the visible granulation tissue was vivid red in colour, and there were no clinical signs of infection. The contact layer of the dressing could be easily removed from the wound surface. The wound was determined to be healed by day 35 *via* second intention healing with epithelium (Fig 1D).

